# Engaging the oldest old in research: lessons from the Newcastle 85+ study

**DOI:** 10.1186/1471-2318-10-64

**Published:** 2010-09-17

**Authors:** Karen Davies, Joanna C Collerton, Carol Jagger, John Bond, Sally AH Barker, June Edwards, Joan Hughes, Judith M Hunt, Louise Robinson

**Affiliations:** 1 Institute for Ageing and Health, Newcastle University, Campus for Ageing and Vitality, Newcastle upon Tyne, NE4 5PL, UK; 2 Institute of Health and Society, Newcastle University, Baddiley-Clark Building, Richardson Road, Newcastle upon Tyne, NE2 4AX, UK

## Abstract

**Background:**

Those aged 85 and over, the *oldest old*, are now the fastest growing sector of the population. Information on their health is essential to inform future planning; however, there is a paucity of up-to-date information on the oldest old, who are often excluded from research. The aim of the Newcastle 85+ Study is to investigate the health of a cohort of 85-year-olds from a biological, medical and psychosocial perspective. This paper describes the methods employed for the successful recruitment, retention and evaluation of this cohort.

**Methods:**

Participants were all individuals born in 1921 and registered with a participating general practice in Newcastle and North Tyneside, UK. Involvement comprised detailed health assessments, by a nurse, in their usual place of residence and/or review of their general practice medical records.

**Results:**

Of the 1453 individuals eligible to participate, 72% (n = 1042) were recruited; 59% (n = 851) consented to both health assessment and review of general practice records. Key factors for successful involvement included protected time to engage with family and other key gatekeepers, minimising participant burden, through for example home based assessment, and flexibility of approach. Cognitive impairment is a significant issue; due consideration should be given to the ethical and legal issues of capacity and consent. Interim withdrawal rates at phase 2 (18 month post baseline), show 88 out of 854 participants (10%) had withdrawn with approval for continued use of data and materials and a further 2 participants (0.2%) had withdrawn and requested that all data be destroyed. Attrition due to death of participants within this same time frame was 135 (16%).

**Conclusion:**

Our recruitment rates were good and compared favourably with other similar UK and international longitudinal studies of the oldest old. The challenges of and successful strategies for involving, recruiting and retaining the oldest old in research, including those in institutions, are described to facilitate adequate representation of this growing population in future research into ageing.

## Introduction

Those aged 85 and over, the *oldest old*, are now the fastest growing sector of the population [1]. Policy and care provision for this age group needs to be informed by reliable estimates of disease prevalence and cognitive and functional impairment, relevant risk factors, the organisation and effectiveness of care services and the changing nature of informal care networks. Conducting detailed research involving the oldest old presents some unique challenges [2]. This group includes many potentially vulnerable individuals with cognitive, functional or sensory impairment and who may be housebound. Around 20% have significant cognitive impairment [3]; it is important to include these individuals in research so as to ensure a representative sample. In England and Wales, the introduction of the Mental Capacity Act [4] enshrined in law consent procedures to be followed where individuals lack capacity.

In the Newcastle 85+ Study, a detailed investigation of clinical, biological and psychosocial factors associated with healthy ageing [5], we found it necessary to devise and validate novel recruitment and consent procedures in order to secure as high a level as possible of ethically sound and productive engagement with members of the oldest old. In this paper, we report some of the challenges of, and successful strategies for, involving, recruiting and retaining this important age group in research studies.

All documents, where stated (see '..' * supplementary information and Newcastle 85+ website) in this paper, can be found on The Newcastle 85+ Study website; http://www.ncl.ac.uk/iah/research/programmes/85plus.htm

### The Newcastle 85+ Study protocol and recruitment

The target population for the Newcastle 85+ Study was all surviving adults born in 1921, who turned 85 in 2006 when the study commenced, permanently registered with a participating general practice in Newcastle or North Tyneside Primary Care Trusts. Participation entailed a detailed multidimensional health assessment, comprising questionnaires delivered on tablet laptop, measurements, function tests and fasting blood sample and/or review of general practice medical records [5]. The health assessment was conducted by a research nurse in the participant's usual place of residence (home or institution); data collection was spread over three main interviews with an additional short visit to measure weight and body composition and collect a fasting blood sample. Together, the three main interviews lasted a mean (standard deviation) of 206 (55) minutes. Recruitment and baseline assessment took place over a 17 month period (2006-2007) with follow up assessments conducted at 18 months (November 2007-May 2009) and 36 months (June 2009-November 2010) post baseline. Participants continue to be followed up if they move into institutional care during the study.

**Figure 1:**
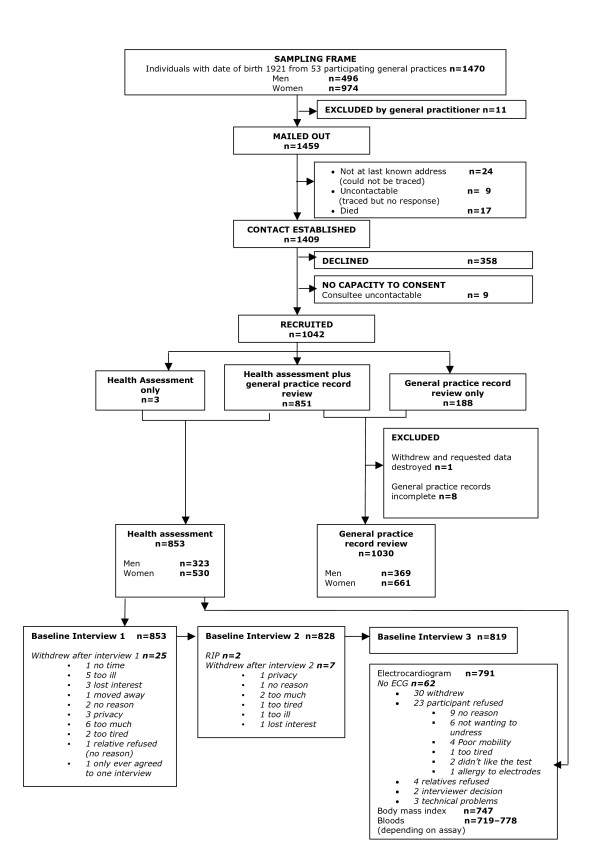
**Recruitment Profile for Newcastle 85+ Study**.

Figure 1 summarises the recruitment of the study cohort [5]. Overall our study recruited 72% (n = 1042) of potential participants with 851 (59% of those eligible) recruited to health assessment plus review of general practice records, and an additional 188 (13%) to record review only and 3 (0.2%) to health assessment only. Of the 64 general practices in Newcastle and North Tyneside Primary Care Trusts, 83% agreed to participate; practices who participated (n = 53) were similar to those who did not (n = 11) [5]. For those practices refusing participation: 4 stated that the surgery was 'too busy' and 7 provided no reason. The recruitment of general practices was undertaken sequentially. Within the target age group, the mortality risk is high [6]. Since a recruitment approach to a deceased person can cause considerable distress to the family, once a general practice had made a final check of their patient list, recruitment letters were posted out within 24 hours. Despite this, in 17 cases when researchers attempted to make contact one week after mail out, they were informed that the individual had died. Therefore, in addition to the precautions taken to avoid such situations, research nurses needed to be trained in how to handle this situation in order to minimise further distress to the families.

### Initial approach and information provision

In some countries including the UK, it is recommended that the initial approach to potential participants in a research study should come from a health or social care professional known to them. In a pilot study for the Newcastle 85+ Study involving around 100 participants, the initial invitation letter to potential participants was sent from their General Practitioner (GP). However, this approach could cause confusion as several potential participants then contacted their general practice surgery with queries about the study, resulting in unnecessary workload for the practice staff. With ethics committee guidance and approval, the approach was therefore modified in the main study, when the initial invitation letter was sent directly from the research team, with an accompanying letter of support from the individual's general practice, together with a detailed information booklet. All written information was prepared using a clear font with a font size of at least 14 point, simple language and short sentences. The use of colour and pictures was employed to make the information booklets more visually appealing. For those with visual impairment, a larger font size or audio-recorded information was available and all study information was made available in other languages, if required.

Older people in particular may be suspicious of research and investigators, perhaps due to feeling vulnerable [7]. Including named photographs of the research nurses within the information booklet for the Newcastle 85+ Study proved valuable, with participants reporting this to have been reassuring to have prior to their first assessment. The initial invitation letter also stated that a named research nurse would telephone them, or visit them, at their home to discuss the study in more detail. A period of one week was allowed before this took place. As in previous research [8-10], and from our own experience, we found that, for the oldest old, the method of direct contact by telephone or home visit to follow up the initial letter was a more successful way of finding out whether individuals were interested in participating in the study. Relying on participants contacting the research team using a reply slip was not utilised, as in a previous study, also recruiting the oldest old, a change in recruitment strategy was requested and approved, after the team posted out 2488 letters with reply slip and received only a 30% (n = 734) response rate from potential participants as to whether or not they wanted a follow up call. For those individuals who agreed to participate in either health assessment and review of general practice records or review of general practice records, only 5% (n = 50) were recruited from home visits with 96% (n = 990) being recruited by telephone contact. Interestingly of individuals who refused any participation in the study, 27% (n = 98) were home visit and 73% (n = 260) were telephone contact *(note 2 participants now withdrawn destroy all and so all of this data is destroyed and not included)*. However, initial approach by telephone and home visit contact does require extra resource. For those participants recruited to health assessment and review of general practice records, up to five telephone calls could be required before any contact was established. Once contact was established, up to nine telephone calls could be required to discuss the study and arrange a convenient time for first appointment. For individuals who were without a telephone it was not uncommon to have two home visits to discuss the study with potential participants and relatives before including any visit to take consent and complete interviews. If the individual resided in a care home (nursing or residential), the total of home visits before consent could be as many as five. For obvious reasons a balance must be struck over how much time should be spent in continuing attempts to make contact. The contact protocol for the Newcastle 85+ Study was developed to try to achieve an appropriate balance (see 'Recruitment/contact protocol' in *supplementary information and on Newcastle 85+ website).

### Engagement of family members in the recruitment process

Family members can play an important gatekeeper role when trying to engage older people in research. Older people are quite likely to request a family member to be present at the initial, and possibly subsequent, assessments before they agree to participate in research. The Newcastle 85+ study team encouraged this and arranged appointments at a time when the chosen family member could be present. However, we found that family members sometimes appeared unduly protective and tried to override the participant's decision to participate; such cases needed be handled with great sensitivity on an individual basis. The research nurses were trained to involve a senior member of the team, usually the research nurse manager or GP member of the study team, as soon as they became aware of any such potential conflict. The need for sensitivity was often greater in care homes where it may be that the desire to protect an older person is mingled with feelings of family guilt and concern as to what activities are appropriate for the older person to participate in.

### Approach to older people living in care homes

The Newcastle 85+ pilot study confirmed that recruiting participants from care homes (nursing and residential homes) was a complicated process, requiring extra time to negotiate with an additional layer of gatekeepers, care home staff [11,12]. In addition, around 60% of people living in care homes have dementia [13] which has implications for consent procedures. In recruiting older people from care homes, it has been argued that if researchers followed all the governance and ethical processes currently required it would be difficult to secure a level of consent/assent above 42% [14].

In the main Newcastle 85+ Study 10% of the sample recruited to health assessment lived in care homes; this figure compares favourably with the 2001 National Census for Newcastle/North Tyneside (12%) and for England and Wales (11.2%). Successful recruitment from care homes was achieved through devising a separate recruitment protocol for people residing in care homes with the specific aim of actively engaging and involving the staff working within them (see 'Recruitment Protocol (own residence)' and 'Recruitment protocol (care home: nursing/residential) in *supplementary information and on Newcastle 85+ website ). Meetings with the care home manager and staff took place to inform them about the relevance of the study and copies of the study documentation were also provided for staff. In addition, since it was recognised that care home staff could not release contact details of clients' relatives directly, copies of information for families were left with care staff with pre-paid envelopes to be sent on if appropriate or legally required (where reduced mental capacity was a factor). It was of course also essential that the older person's free will and privacy not be ignored; all letters addressed to them were sealed and delivered directly.

### Assessment of capacity and informed consent

Significant cognitive impairment may be present in around a fifth of older people over the age of 85 [3] and due consideration must been given to the ethical implications of involving cognitively impaired people in research. The Mental Capacity Act specifies that researchers assume capacity of all individuals regardless of age, appearance or behaviour[4]. Incapacity must be demonstrated through assessment whereupon consent/consultee opinion should be sought. Researchers are also required to follow specific guidance and seek consultee opinion should they be made aware of incapacity during the lifetime of the study.

#### i) Assessing capacity to give consent

As practical guidance on the implementation of the Mental Capacity Act in research settings was limited when it was first introduced, we developed comprehensive consent protocols and documentation to which research nurses were required to adhere. These protocols were then expanded upon and terminology corrected to incorporate all of the requirements of the Mental Capacity Act as detailed evidence became available. In consultation with a Consultant Old Age Psychiatrist, who was also an expert in ethical issues, we developed a consent pathway allowing the research nurse to ascertain step by step whether an individual had capacity to understand the nature and demands of the study. The pathway assesses the older person's ability to understand, retain and use the information to make a decision and the ability to communicate that decision. Within our consent protocol alerts were built in instructing the nurses to consult with senior members of the research team should the consent process become problematic (see 'Consent Protocol' and 'Consent Pathway' in *supplementary information and on Newcastle 85+ website).

A consent checklist was also devised for the nurses to work through before any consent documentation was signed off (see 'Consent Checklist' in *supplementary information and on Newcastle 85+ website). Due to the complexity of this area, all researchers working with this group were required to undergo formal training, with regular updates, in the process of assessing capacity and obtaining valid informed consent. It was made clear to participants, their relatives and carers that consent could be withdrawn at any time without reason and was not legally binding. A copy of the signed consent form was provided to participants.

#### ii) Securing consent if the person lacked capacity at onset of the study

For those individuals who lacked capacity to give full informed written consent, we first sought an opinion independently from an appropriate personal consultee (formerly referred to as proxy), namely their next of kin, immediate carer or attorney with Lasting Power of Attorney, who made the final decision. The identified consultee was given full information about the study, why they had been chosen as a consultee and their responsibility as consultee (see 'Consultee Information booklet' in *supplementary information and on Newcastle 85+ website). They were asked to use their knowledge of the participant, past and present, to determine whether in their opinion, the potential study participant would have had no objection to entering the study when cognitively intact, and that they would not be caused undue distress by participation (see 'Consultee Opinion Form' in *supplementary information and on Newcastle 85+ website).

If the individual approached was not willing or indeed able (e.g. lacked capacity themselves) to act as a personal consultee, or if no close relative or friend had been identified, the introduction of the Mental Capacity Act enabled the research team to consider all other relevant social/care networks to decide who might be suitable to approach as a nominated consultee [4]. This would be someone with a professional relationship to the individual but who held no connection to the research. It is also worth noting that these same professional individuals are a valuable additional resource to researchers when called upon to act as informants, as often they are best placed to provide detailed information. In such cases, it is recommended as courtesy that the researcher discuss with the official consultee the benefit of using an additional informant. In the baseline phase of the Newcastle 85+ Study, in nine cases contact was not established with an appropriate consultee despite repeated attempts; each case was brought to the research nurse manager or GP member of the study team and a decision made as to when to abandon recruitment. Had this legislation been in place during baseline recruitment it is possible that the nine participants lost due to 'consultee uncontactable' (see Figure 1), would have been recruited as the main obstacle here was the uncertainty as to whether individuals from other social/care networks could act as 'consultee'. However, any consultee opinion would not take priority over a refusal, either verbal or non-verbal, from the participant during any element of the research study; an example here might be reluctance to hold out their arm when drawing blood.

The very act of locating an appropriate personal or nominated consultee was not without problems. The next of kin might not respond to or return calls or consider research as a high priority, there may also be feeling of guilt or complex family dynamics, which must be met with great care and respect. As suggested earlier, if the older person is in a care home setting, then first contact with family i.e. personal consultee must be established through care home staff or in some cases general practice staff. There may be reluctance from individuals or official bodies to act as a nominated consultee and again researchers must demonstrate that they have taken steps to approach the most appropriate party to represent the views of the participant. Of those who agreed to the health assessment, 11% (98/854) legally required consultee opinion, of whom 64% (63/98) resided in an institution. For 29% (247/854) of the health assessment participants, an informant supplied information in at least one of the interviews.

#### iii) Loss of capacity to consent during the research study

It is important to note that in such an age group, capacity can temporarily be lost, for example due to toxicity from a urine or chest infection. In such cases, research activity was suspended until capacity returned. If however there was a permanent loss of capacity, for example due to dementia, then consultee opinion was required not only for continued participation but also for continued use and storage of data material already gained (see 'Consultee Retrospective Approval Form for Loss of Capacity' in *supplementary information and on Newcastle 85+ website). This underlines the importance of adopting the principle of "process consent", which requires confirmation of consent at each appointment and within each appointment of the study allowing for changes in participant preferences and changes in capacity [4]. This is in contrast to clinical trials of medicinal products, where consent from an adult to participate in the trial remains valid, even after loss of capacity, provided the trial is not significantly altered [15].

In our participant consent document, we included a request for the participant to nominate an appropriate personal consultee with whom researchers could liaise, should they lose capacity during the course of the study. The rationale for nomination of consultee was provided in full to study participants as an integral part of the participant information booklet (see 'Participant Information Booklet' in *supplementary information and on Newcastle 85+ website), and additional verbal information was provided at the time of consent. Consent documents also included seeking participants' opinion as to whether they wished to remain in the study and whether they would allow previously collected data to be used in the analysis, should they lose capacity (see 'Participant Consent Form' in *supplementary information and on Newcastle 85+ website).

### Interviewing the oldest old: minimising participant burden

The oldest old are less likely to have their own transport and any need to travel to a research venue can exclude this age group from participation. There is evidence from randomised trials that the distance between study site and the person's residence affects participation, with those at greater distance being less likely to participate[16]. Despite the extent of detailed clinical and functional data we sought to collect, we conducted all health assessments in participants' homes. Previous research had shown this is inevitably more expensive, but that it enhanced recruitment rates in a very old population [17]. In the Newcastle 85+ pilot study, more than half of those recruited said they would not have participated if they had been required to attend hospital or other clinical settings (unpublished data).

The content of our interview schedule and its duration were the subject of much discussion within the study team. Our experience and information from previous studies have shown that it can take up to four times longer to acquire information from older people [18]; and that older people may develop fatigue during an assessment. Therefore the organisation of questions within the interview is important [2]; the most relevant should be placed first but always within limits of what is acceptable and sensitive. We felt it reasonable that a single interview session should last no longer than 90 minutes; our lengthy assessment schedule was split into three interview sessions conducted around a three week period, plus an additional visit to obtain blood samples. Questionnaire sections were prioritised between and within interviews based upon importance, time to complete and a common sense approach to an appropriate order from a participant's perspective. For some, fatigue was still a problem and therefore interviews were further split into several shorter interviews. For more active participants, problems were experienced with fitting assessments into busy calendars. It was important never to assume that an older person cannot attempt a task purely because of his or her age. Whenever the study involved the use of instruments validated in general populations, these were tested and considered in relation to older people in order to identify any particular areas of difficulty and the time needed to complete them. In our experience the use of tablet laptop to capture data did not place any additional burden on participants or create a barrier to dialogue and interaction between the research nurse and participant.

### Retention of study participants: interim withdrawals

As part of our 'Withdraw from study protocol' participants were informed that they could withdraw at any time from all or part of the research. In addition they were told that they could suspend research activity for a period of time should they wish to do so, with the researcher contacting the participant or consultee after this period of time to review the situation. Withdrawal rates between baseline and phase 2 (18 month post baseline), show 88 out of 854 participants' (10%) had withdrawn with approval for continued use of data and materials and a further 2 participants (0.2%) had withdrawn and requested that all data be destroyed. The commonest reasons for withdrawal were deterioration in health ((24 (29%), with 17 of these individuals subsequently being reported as deceased) and fatigue (14 (17%)). This is similar to previous studies which found that attrition of older people from research for reasons other than death is only related to a small number of factors [19,20]. Attrition due to death of participants within this same time frame was 135 (16%).

### Benefits and risks for participants and researchers

We were careful in planning the study to consider and explain any potential benefits to older people of research participation. These included altruistic reasons, i.e. for the greater good and to help others; such reasons tend to predominate in studies involving both older adults and people with existing long term conditions [21]. In addition, participants often appreciated the social opportunities provided by the interviewer visits [2]. In the Newcastle 85+ Study, participants often made comments such as "I am just glad I can be of help", "I look forward to your visit" and "I enjoy the company".

Researchers have a responsibility to ensure that engagement continues in an appropriate manner. It is necessary to remember that the research relationship is based upon trust and this should not be misrepresented in order to coerce an older person to participate or remain in research. The process of cessation of the study needs to be carefully thought through, so as not to leave the older person feeling bereaved or used in any way [22]; the study information booklet needs to state clearly the level of participant involvement. It is also necessary to plan the provision of ongoing communication about the study, in the form of face-to-face and written feedback, together with Christmas cards as a means of keeping in touch and thank you letters and certificates after participants have completed assessments.

The Newcastle 85+ Study did not include any trial of therapy therefore risks to participants were minimal but did include the possibility of falls during physical function tests and occasional bruising and discomfort from blood taking. The option of a chaperone was always offered to participants if the gender of the interviewer was different to theirs and also if some intrusive procedure was to be undertaken such as a domiciliary echocardiogram. This was felt to provide safety and/or reassurance to the participant. Local police were informed about the study and potential participants and their representatives were provided with a police contact number should they wish to check the legitimacy of the study. Research nurses visiting participants' carried University photo identity cards in addition to study identity cards using the same photograph as included in the participant information booklet.

The safety of the nursing research team must also be considered, as they are lone workers within the community dealing with a vulnerable group. Often older people are viewed in negative terms as passive, frail individuals but they may become agitated or aggressive, as may their relatives and pets! All researchers undertaking community visits received safety training and were required to sign a safety protocol (see 'Staff Undertaking Domiciliary Visits' Safety Protocol' in *supplementary information and on Newcastle 85+ website), a mobile telephone was provided and all visits, together with their estimated duration, were logged with an automated call back facility which checked in with the interviewer when the estimated time had elapsed. If the interviewer did not respond to two prompts then an automatic trigger of alerts to senior team members was raised. In addition, chaperones were available to the study team during 'out of hours' visits, or in perceived problem localities where staff might feel vulnerable. Staff were also provided with a car breakdown service, breakdown sign and personal alarms.

### Public and patient engagement in research design

During the pilot study and the development of the main Newcastle 85+ Study, extensive consultation took place with older peoples' consumer groups such as Age Concern, together with other stakeholders including Primary Care Trusts; Social Services; GPs; community nursing staff and care home managers. These groups helped with the design of participant literature, the recruitment strategy and the content of the assessment protocol. In addition, detailed feedback was obtained from the pilot study participants about their experiences, which proved very helpful in planning appropriate scheduling and timing of assessments, the clarity of research documentation and the usefulness and design of prompt cards.

Wider methods were employed to publicise our study and to engage with relevant stakeholders as fully as possible. A study launch event, to which key stakeholders were invited, helped raise the study profile. Posters and information leaflets were distributed to GP surgeries, articles published in Primary Care Trust newsletters and members of the project team gave presentations to key local groups and voluntary organisations. The media has also been shown to have a role in facilitating involvement of older adults in research [23]; The Newcastle 85+ study team organised local news coverage through television and newspapers which proved successful as many individuals who thought they met the inclusion criteria subsequently contacted the study team directly.

## Conclusion

In order to guarantee that the future care provided to our ageing populations is grounded in the best quality evidence, it is imperative that researchers ensure their study populations are representative and include the oldest old, the most rapidly expanding sector of the population. Older people, especially those over 75 and those in care homes, are often unjustifiably excluded from studies [24-26]; this must be addressed by both the funding commissioners and the research community by tackling cultural/investigator bias and unjustified age limits. Our recruitment rates were good considering the age of the sample and the extensive assessment involved. These rates also compare favourably with other UK longitudinal studies of ageing, which have often involved younger cohorts [3,27], and with similar studies internationally [28-30]. We accept that variation in local circumstances may be an important factor and that the procedures used here were suitable for the particular population under study and may need suitable modification for other populations. Although this is a limitation of the study, we feel that researchers in other areas will readily appreciate the need to modify procedures where necessary.

Our experiences with residents of care homes confirmed previous recommendations [12,14,31] that the additional time required to recruit this very important sub group of older people is substantial. As Zermansky et al. (2007)[14] commented, "conducting research with care home residents is beset with constraints and complexities that can impair progress.....an extraordinary amount of time and resources are needed to overcome them". It is therefore essential that such resources be costed into the project funding and that, in order to inform these costings, procedures for recruitment from care homes be tested in a pilot study.

Throughout this paper, we have outlined issues we encountered in involving and retaining the oldest old in a large cohort study of health and ageing in one area of the UK and put forward approaches we found useful to successfully address these. In summary, important factors to consider when engaging the oldest old in research include:

**• Initial participant approach**. An initial invitation from a known health care professional may lead to more confusion than a letter from the study team. Photographs of researchers are useful additions to study information sheets. Adequate time for recruitment and data collection should be factored into planning.

**• Engagement with family and care home staff**. Additional time and resources are needed, especially with the oldest old, to engage with family carers and other gatekeepers such as care home staff for those people living in care. Researchers should be trained and supported to ensure that complex situations are handled with sensitivity.

**• Significant cognitive impairment is an issue; due consideration must been given to the relevant ethical and legal issues, including assessment of capacity and consent procedures**. The use of protocols to assess capacity and to secure consent should be developed. Training for researchers in the assessment of capacity and seeking informed consent should be mandatory.

**• Minimising participant burden**. Home visits are preferred by this population and may enable the participation of those who are frail or housebound. Consequently researchers' travel costs, specialised portable equipment and availability of chaperones may need to be costed into the research. The need for, and the cost of, several, shorter visits to assess capacity, secure consent and collect all relevant data should be anticipated.

**• Participant withdrawal**. Researchers should endeavour to make the research process as flexible as possible and may wish to consider the option of study suspension as an alternative to withdrawal as often the participant does not wish to withdraw permanently but cannot accommodate study visits at that particular time due to other commitments.

**• Communication with participants after study cessation**. The oldest old may derive benefits, such as opportunities for increased socialisation, from participation in research. The process of study close-out should be considered at the onset so that participants are not left with a feeling of loss. A communication strategy to update participants about the study progress and findings after their involvement ceases can be helpful.

**• Engagement with stakeholders and the media**. Researchers should consider the need to engage with not only local clinicians, but also with older people's voluntary organisations, the media and the local press. This should be proactively built into the study recruitment protocol.

## Competing interests

The authors declare that they have no competing interests.

## Authors' contributions

KD Research Nurse Manager, developed the research protocols and contributed to the development of questionnaires; Lead joint author and corresponding author. Initial conception of paper, identification and review of relevant literature, writing up of paper, critical comments on drafts of paper. Final manuscript read and approved. JC Project manager, developed the questionnaires and research protocols, advice on design of paper, significant input into and critical comments on drafts of paper. Final manuscript read and approved. CJ Principal investigator on 85+ study, input to development of questionnaires advice on design of paper, significant input and critical comments on drafts of paper. Final manuscript read and approved. JB Principal investigator on 85+ study, input to development of questionnaires and advise on fieldwork issues; Advice on design of paper, significant input and critical comments on drafts of paper. Final manuscript read and approved. SB Research Nurse; Contributed to the acquisition of data, provided input to literature review. Critical comments on drafts of paper. Final manuscript read and approved. JE Research Nurse; Contributed to the acquisition of data, provided input to literature review. Critical comments on drafts of paper. Final manuscript read and approved. JH Research Nurse; Contributed to the acquisition of data, provided input to literature review. Critical comments on drafts of paper. Final manuscript read and approved. JH Research Nurse; Contributed to the acquisition of data, provided input to literature review. Critical comments on drafts of paper. Final manuscript read and approved. LR Senior Investigator on 85+ study; Joint lead joint author. Initial conception of paper, identification and review of relevant literature, writing of paper, critical comments on drafts of paper. Final manuscript read and approved. All authors read and approved the final manuscript.

## Pre-publication history

The pre-publication history for this paper can be accessed here:

http://www.biomedcentral.com/1471-2318/10/64/prepub
